# Comparative genomics of two Vietnamese* Helicobacter pylori* strains, CHC155 from a non-cardia gastric cancer patient and VN1291 from a duodenal ulcer patient

**DOI:** 10.1038/s41598-023-35527-4

**Published:** 2023-05-31

**Authors:** Bui Hoang Phuc, Vo Phuoc Tuan, Tran Thanh Binh, Pham Huu Tung, Tran Dinh Tri, Ho Dang Quy Dung, Ngo Phuong Minh Thuan, Kartika Afrida Fauzia, Evariste Tshibangu-Kabamba, Ricky Indra Alfaray, Batsaikhan Saruuljavkhlan, Takashi Matsumoto, Junko Akada, Yoshio Yamaoka

**Affiliations:** 1 Department of Environmental and Preventive Medicine, Oita University Faculty of Medicine, Yufu, Oita Japan; 2 Faculty of Applied Technology, Van Lang University, Ho Chi Minh City, Vietnam; 3 Department of Endoscopy, Cho Ray Hospital, Ho Chi Minh City, Vietnam; 4 Department of Public Health and Preventive Medicine, Faculty of Medicine, Universitas Airlangga, Surabaya, 60115 Indonesia; 5 Helicobacter pylori and Microbiota Study Group, Institute of Tropical Disease, Universitas Airlangga, Surabaya, 60115 Indonesia; 6 Department of Parasitology, Graduate School of Medicine, Osaka Metropolitan University, Osaka, Japan; 7 Research Center for GLOBAL and LOCAL Infectious Diseases, Oita University, Yufu, Oita Japan; 8 Department of Medicine, Gastroenterology and Hepatology Section, Baylor College of Medicine, Houston, TX USA

**Keywords:** Cancer, Microbiology, Gastroenterology, Pathogenesis

## Abstract

*Helicobacter pylori* is involved in the etiology and severity of several gastroduodenal diseases; however, plasticity of the *H. pylori* genome makes complete genome assembly difficult. We report here the full genomes of *H. pylori* strains CHC155 and VN1291 isolated from a non-cardia gastric cancer patient and a duodenal ulcer patient, respectively, and their virulence demonstrated by in vitro infection. Whole-genome sequences were obtained by combining long- and short-reads with a hybrid-assembly approach. Both CHC155 and VN1291 genome possessed four kinds of genomic island: a *cag* pathogenicity island (*cag*PAI), two type 4 secretion system islands within an integrative and conjugative element (*tfs* ICE), and prophage. CHC155 and VN1291 carried East Asian-type *cagA* and *vacA* s1m1, and outer membrane protein genes, including two copies of *oipA*. Corresponded to genetic determinants of antibiotic resistance, chromosomal mutations were identified in CHC155 (*rdxA, gyrA*, and *23S rRNA*) and VN1291 (*rdxA*, *23S rRNA*, and *pbp1A*). In vitro infection of AGS cells by both strains induced the cell scattering phenotype, tyrosine phosphorylation of CagA, and promoted high levels of IL8 secretion, indicating fully intact phenotypes of the *cag*PAI. Virulence genes in CHC155 and VN1291 genomes are crucial for *H. pylori* pathogenesis and are risk factors in the development of gastric cancer and duodenal ulcer. Our in vitro studies indicate that the strains CHC155 and VN1291 carry the pathogenic potential.

## Introduction

*Helicobacter pylori* is a Gram-negative bacteria that chronically infects the human stomach and in 2015 there were approximately 4.4 billion individuals worldwide infected with *H. pylori*^1^. *H. pylori* infection is strongly associated with gastric neoplasm and 1–3% of such cases will develop gastric cancer compared with 0% in uninfected patients^2–5^. Vietnam has one of the highest *H. pylori* infection rates in Asia (70.3%)^1^ and a high age-standardized rate (ASR) of stomach cancer prevalence (15.5)^1,6^ according to GLOBOCAN 2022^7^. However, there is minimal knowledge of *H. pylori* genomes from this region, as well as the prevalence of virulence factor (CagA variable region) and drug-resistance genes, which limits the application of molecular diagnostics, such as tests for resistance genes, and the development of vaccines and therapeutic drugs^8–11^.

In addition to host and environmental factors, *H. pylori* virulence factors play a crucial role in increasing the severity of clinical outcomes^12^. The *H. pylori* genome is approximately 1.6 Mbp and contains more than 1600 genes. Each strain harbors a unique subset of genes that help adaptation to the human gastric environment^13^. Virulence factor genes are protein coding. Some are essential for bacterial survival in acid environments (e.g. *ure* genes encode urease), some encode outer membrane proteins that promote adherence to gastric epithelial cells (*hopQ*, *oipA* and *babA*), and some activate the innate immune response. CagA plays a crucial role in *H. pylori* gastric pathogenesis by being injected into host cells and disturbing internal signaling. VacA is secreted into the gastric environment and can enter the cytosol of host cells where it forms vacuoles associated with apoptosis^13–15^. Therefore, identifying sets of virulence factor genes from strains isolated from gastric cancer and duodenal ulcer patients will help to elucidate the virulent strains of *H. pylori*. Virulence factors of *H. pylori* have recently been classified according to functional categories, including acid resistance, adherence, chemotaxis and motility, molecular mimicry, immune invasion and modulation, secretion system, proinflammatory effect, and endotoxin production^16^. The pathogenicity of *H. pylori* is dependent on virulence factors that help bacteria invade gastric epithelial cells, change cell morphology, and induce the host immune response through an inflammatory pathway. These effects can contribute to gastroduodenal diseases^17^. Furthermore, long-term *H. pylori* infection is considered a high risk for the development of gastric cancer^2^.

Genomic islands are clusters of genes within a bacterial genome that appear to have been acquired by horizontal gene transfer. Many genomic islands are flanked by repeat sequences and carry fragments of or complete mobile and accessory genetic elements, such as bacteriophages, plasmids, insertion sequence elements, and integrative and conjugative elements (ICEs)^18,19^. Genomic islands are common in both Gram-positive and Gram-negative bacteria and play diverse roles in adaptation, metabolism, fitness cost, increased virulence, and antibiotic resistance^18^. In *H. pylori*, the *cag* pathogenicity island (*cag*PAI) is a genomic island that encodes functional components of a type 4 secretion system (T4SS)^20^. This T4SS represents a needle-like structure protruding from the bacterial surface and is induced when it comes into contact with a host cell before injecting oncogenic CagA^20^. When CagA is translocated into the cytosol, it triggers proinflammatory activity and internal signaling cascades^21^. Other genomic islands in *H. pylori* include *tfs3* and *tfs4* ICEs, which both encode novel T4SS components^22,23^. Similar to *cag*PAI, *tfs* ICEs are considered gastroduodenal disease markers^24,25^. In addition, *H. pylori* can contain a prophage, a genome island that originates from a bacteriophage and is found in approximately 20% of *H. pylori* isolates^26^.

We previously performed PCR-based analyses that detected a small fraction of virulence factors^10,27^ but not a full gene or genomic island. Current advances in genome sequencing technology, including shotgun sequencing and long-read sequencing, make it possible to accurately obtain full bacterial genomes. There is plasticity in the *H. pylori* genome; some genes are duplicated (*oipA* in hspEAsia strains) and strains have highly similar gene families, i.e. outer membrane proteins and genome island clusters, which makes complete genome assembly difficult. Individual strain information therefore tends to be lacking. In addition, some strains possess a plasmid, which is rarely detected using only short-read assembly. To overcome these characteristics of *H. pylori* genomes, we used third-generation sequencing to generate long-reads to facilitate complete genome assembly. Complete genomes are useful for further characterization of virulence factors and their phenotypes. Here, we report two complete genomes of *H. pylori* strains, CHC155 and VN1291, isolated from a patient with gastric cancer and from a duodenal ulcer patient, respectively. We identified four genomic islands in each strain and characterized their virulence factors and genetic determinants of antibiotic resistance. These features, supported by phylogenic analysis, were representative of two Vietnamese strains.

## Results

### Clinical characteristics of the patients and features of *H. pylori* strains CHC155 and VN1291

We have previously obtained over 100 *H. pylori* strains from Vietnamese gastric cancer and duodenal ulcer patients^24^. We chose strains CHC155 and VN1291 for this study, because they contain fully intact tfs3 ICEs in addition to *cag*PAI.* H. pylori* CHC155 was isolated from a gastric biopsy specimen collected by endoscopy from a 61-year-old Vietnamese male patient with non-cardia gastric cancer. The clinical isolate showed no in vitro resistance to tetracycline or amoxicillin with minimum inhibitory concentrations (MICs) of 0.12 and 0.06 mg/L, respectively. However, resistance was noted to clarithromycin, levofloxacin and metronidazole, with MICs of 4 mg/L, 2 mg/L, and ≥ 256 mg/L, respectively. Strain VN1291 was isolated from a 43-year-old female patient with duodenal ulcer at Cho Ray Hospital, Ho Chi Minh. This strain was resistant to amoxicillin, clarithromycin and metronidazole, with MICs of 0.5 mg/L, 4 mg/L, and ≥ 256 mg/L, respectively, but was susceptible to levofloxacin and tetracycline, with MICs of 0.5 mg/L and 0.125 mg/L, respectively.

**Figure 1:**
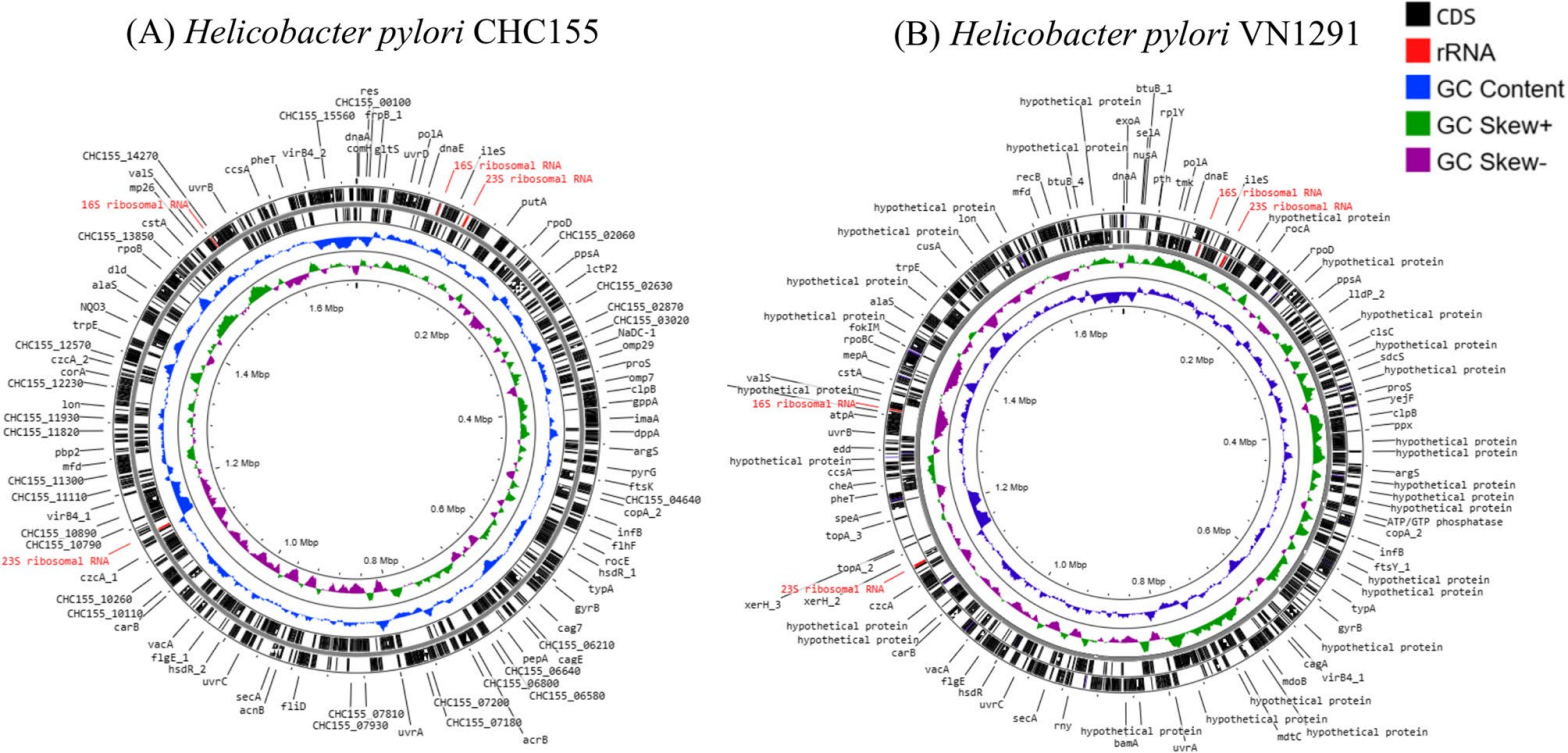
Overview of the genomes of *H. pylori* strain CHC155 (**A**) and VN1291 (**B**). The plot displays the genomes of CHC155 and VN1291 as circular chromosomes of 1,696,601 bp and 1,702,481 bp. The black outermost and innermost rings indicate the genes, which were predicted and annotated using PROKKA v.1.14.6 (https://github.com/tseemann/prokka/). GC skew and GC content are indicated. The genome map of strain CHC155 and VN1291 were visualized from Genbank format by Proksee (https://proksee.ca/)^30^.

The de novo assembly of the CHC155 genome resulted in a single circular contig of 1,696,601 bp. Using the CheckM v1.1.6 algorithm^28^, the genome assembly reached > 99% completeness with no contamination or strain heterogenicity. DFAST quality control in FastANI v.1.33^29^ showed the highest average nucleotide identity of the CHC155 genome to be 94.9% against *H. pylori* strain ATCC43504 (GCA_004295525.1) which was assigned to the same species. Strain CHC155 contained four *rRNA* genes, including two copies of 23S *rRNA* and two copies of 16S *rRNA* (Table 1 and Fig. 1A). We also determined the full genome of strain VN1291 (Table 1 and Fig. 1B), for comparative assessment of CHC155 virulence.

**Table 1:** Genome characteristics of strains CHC155, VN1291, and 26695.

Strain	CHC155	VN1291	26695
Genome size (bp)	1,696,601	1,702,481	1,667,867
GC content (%)	38.5	38.5	38.9
Number of coding sequences	1580	1599	1578
Average amino acid length	325.7	322.8	319.0
Coding ratio (%)	91.0	91.0	90.5
Number of rRNAs	4	4	4
Number of tRNAs	36	37	36
Number of CRISPRs	1	1	0
Genomic island, *cag*PAI	1	1	1
Genomic island, *tfs3* ICE	2	1	1
Genomic island, *tfs 4* ICE	0	0	1
Genomic island, *tfs3_4* hybrid ICE	0	1	0
Genomic island, prophage	1	1	0
References	This study^24^	This study^24^	^31^

We detected one 21-bp CRISPR-like sequence (CTTCAATCAAGGCA^30^CTTATAA) in both strains. This sequence was located in the *vlpC* gene, which encodes a putative vacuolating cytotoxin (*vacA*)-like protein C, an outer membrane protein toxin.

### Genomic island prediction and sequence comparison

**Figure 2:**
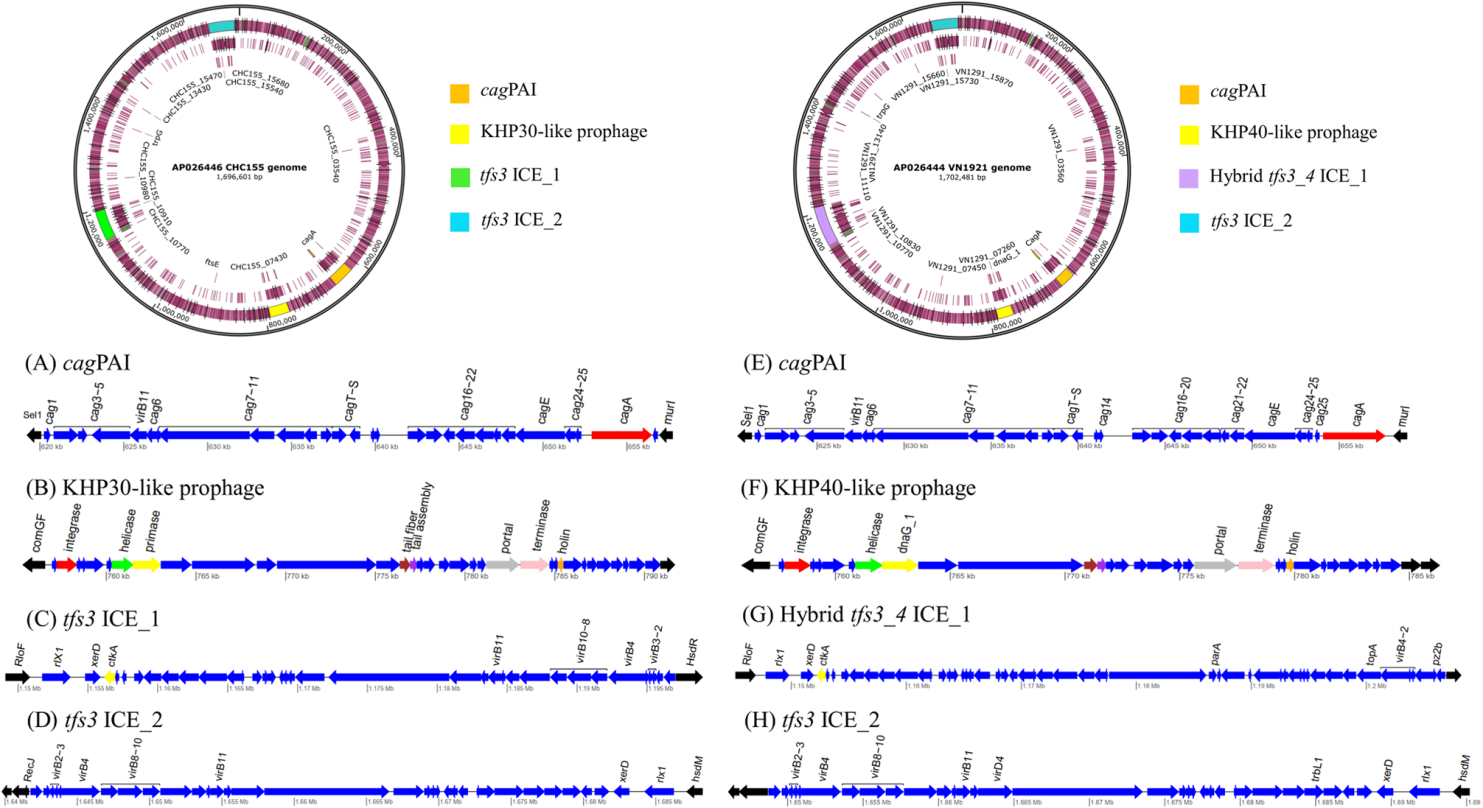
Overview of the genomic islands (GIs) in strain CHC155 and VN1291 genomes. Locations of four genomic islands in both genomes are visualized by color scheme using SnapGene v.6.2.2. The genetic schemes of GIs in two strains (**A**–**H**) were plotted by genoplotR v.0.8.11. (**A**–**D**) GIs in strain CHC155; (**A**) *cag*PAI, (**B**) KHP30-like prophage, (**C**) *tfs3* ICE_1, and (**D**) *tfs3* ICE_2. (**E**–**H**) GIs of strain VN1291; (**E**) *cag*PAI, (**F**) KHP40-like prophage, (**G**) hybrid *tfs3_4* ICE_1, and (**H**) *tfs3* ICE_2. The black color indicates the flanking genes of GIs: *sel1* (left) and *murI* (right) in *cag*PAI, *comGF* (left) and gene encodes DNA-binding protein (right) in KHP30-like prophage, gene encodes RloF protein (left) and *hsdR* (right) in *tfs3* ICE_1, *hsdM* (left) and gene encodes RecJ protein (right) in *tfs3* ICE_2. The red arrow indicates *cagA*, an effector protein of *cag*PAI, and the yellow arrow indicates *ctkA* (cell-translocating kinase A), an accessory gene of *tfs3* ICE. Some genes of interest in KHP30-like prophage and KHP40-like prophage are marked and colored.

GI prediction was based on common features of genomic islands, including mobility genes, phage-related genes, direct repeats, and nucleotide composition bias. Genomic islands identified in strain CHC155 included two *tfs3* ICEs, a KHP30-like prophage, and a *cag*PAI (Fig. 2A). Four genomic islands were identified in strain VN1291, including *tfs3* ICE, hybrid *tfs3*_*4* ICE, a KHP40-like prophage, and a *cag*PAI (Fig. 2B).

1. *cag*PAI

Strains CHC155 and VN1291 both possessed a 30-kb *cag*PAI, a low GC content region (less than 35%) that contained 24 *cag* genes, and a gene coding the effector protein, CagA (Fig. 2 and Supplementary Table S1). The *cag*PAI was inserted between glutamate racemase (*murL/I)* (right) and Sel1-like repeat protein (*sel1*) genes in both CHC155 (Fig. 2) and VN1291 (Supplementary Fig. S2).

2. T4SS within the *tfs* ICE

Strains CHC155 and VN1291 both possessed two *tfs* ICEs (Figs. 1, 2). Based on the genetic arrangement of *rlx (virD2 relaxase)*, *xerD* (integrase), *virB6* (T4SS gene), and sequence identity, the two *tfs* ICEs in strain CHC155 were classified as *tfs3* ICEs (Fig. 2). One *tfs3* ICE (1,141,730–1,197,010) was flanked by the gene for RloF protein (left) and the gene for a type I restriction enzyme R protein (HsdR) (right) (Supplementary Table S1 and Fig. 2C). The flanking genes of the other *tfs3* ICE (1,639,789–1,688,225) were the gene for a type I enzyme M protein (HsdM) (left) and RecJ (right), a single-stranded DNA-specific exonuclease involved in archaeal DNA replication initiation (Fig. 2D and Supplementary Table S1). Based on the genome comparison, strain VN1291 possessed a hybrid *tfs3_4* ICE (1,137,628…1,209,303) that was flanked by genes for a RloF-like protein (left) and a DHH family protein (right) (Fig. 2G and Supplementary Table S1). In addition, the second *tfs3* ICE of strain VN1291 (1,646,779–1,695,191) was flanked by the gene for type I restriction enzyme M protein (HsdM) (left) and a putative metal-dependent hydrolase (right) (Fig. 2H and Supplementary Table S1). All *tfs* ICEs in strains CHC155 and VN1291 contained genes that encode a T4SS: *virB2*, *virB3*, *virB4*, *virB6*, *virB8*, *virB9*, *virB10*, *virB11*, and *virD4*. In addition, they harbored the DNA processing genes, *virD2 relaxase* and *xerT integrase/recombinase* (Fig. 2). In both strains, one *tfs3* ICE contained the cell-translocating kinase A (*ctkA*) gene, a translocation protein of *tfs3* ICE. In addition, the longest *tfs3* ICE gene was an 8600 bp DNA methyltransferase.

3. Prophage

We identified a KHP30-like prophage (encoding 34 phage proteins) in strain CHC155 and a KHP40-like prophage (encoding 35 phage proteins) in strain VN1291 (Table 2). The 5′-*attR/attL-*3′ repeat region (ATTTTTAAAATA) was identified in the prophages of both CHC155 and VN1291. The KHP30-like and KHP40-like prophages were integrated between the competence protein, *comGF* (left), and a putative outer membrane protein (right) (Fig. 2). These prophages encoded proteins that play roles in host cell invasion, including integrase, tail, portal, holin, and terminase proteins. Other proteins were phage-related hypothetical proteins (Supplementary Table S1).

4. Phylogenomic analysis of strains CHC155 and VN1291

**Table 2:** Prophage characteristics in strains CHC155 and VN1291.

Strain	CHC155	VN1291
Length (kb)	34.8	28.7
Completeness (score)	Intact (145)	Intact (140)
Position	755,351–791,683	757,548–786,311
*att* site	Yes	Yes
No. of phage proteins	34	32
Attachment site	Yes	Yes
Most common phage	KHP30^32^	KHP40^32^
GC%	36.48%	36.18%

**Figure 3:**
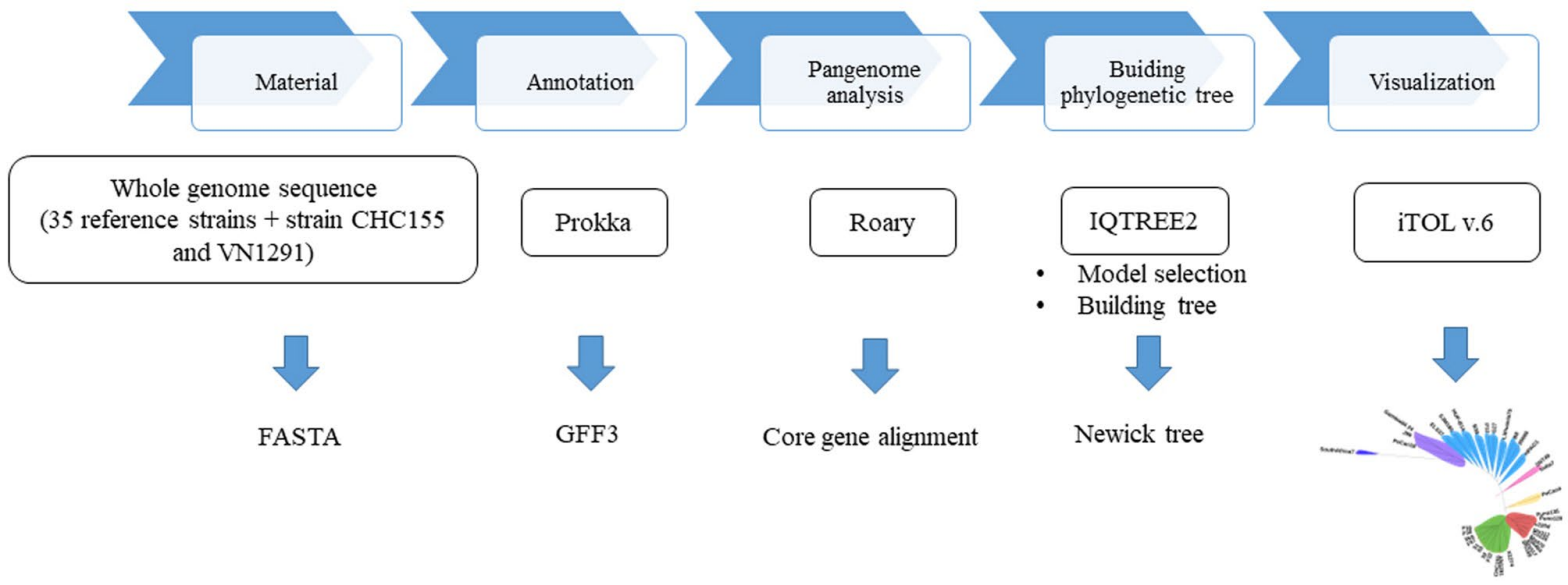
Flow diagram for phylogenomic analysis of *H. pylori* CHC155 and VN1291. Whole-genome sequences of strains CHC155, VN1291, and 35 reference strains were annotated by Prokka. Subsequently, the genome feature (.gff) pangenome was analyzed by Roary and exported as a core gene alignment. Using this alignment, the phylogenetic analysis was performed and exported as a Newick tree, which was visualized in a radical image. The flow diagram was drown in PowerPoint (Office 365, https://www.microsoft.com/vi-vn/microsoft-365).

**Figure 4:**
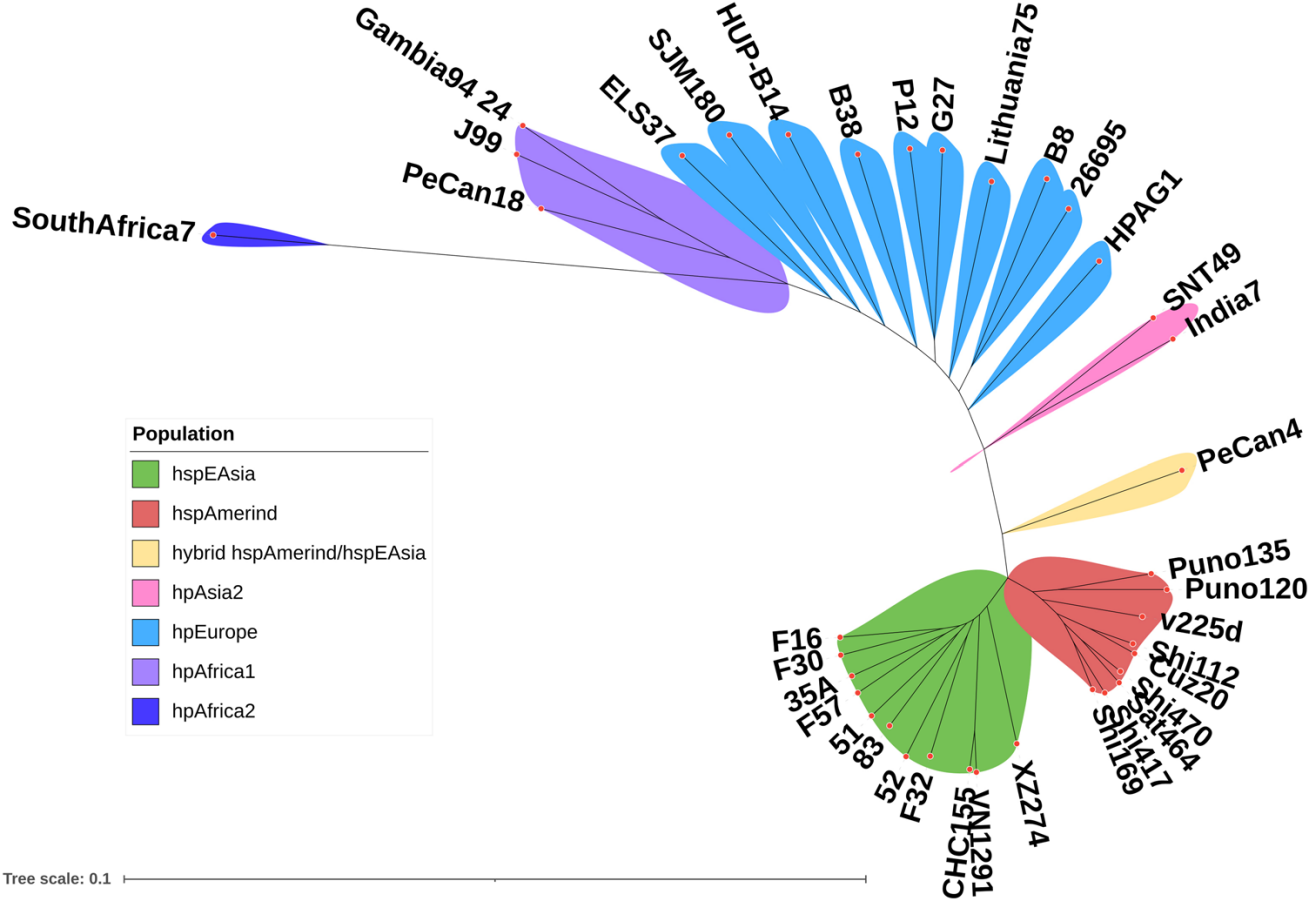
Phylogenetic tree constructed from core single nucleotide polymorphisms of *H. pylori* CHC155, VN1291, and 35 reference strains, which were representative of different geographical populations. The tree map was visualized by iTOL v.6.7.3 (https://itol.embl.de/). The Newick format was generated from IQTREE2, *H. pylori* population was annotated into different colors.

The phylogenomics of strains CHC155 and VN1291 were analyzed according to the flow diagram in Fig. 3. Pangenome analysis using Roary identified 3330 genes among 37 *H*. *pylori* strains, which included 1036 conserved genes (32.8%) present in 99% of all strains (Supplementary Table S7). IQ-TREE identified 975,924 sites from the core gene alignment. The total number of sites divided into 130,962 parsimony-informative (13.4%); 77,543 singleton (8.0%), and 767,419 (78.6%) constant sites. ModelFinder selected the best fit model of substitution (GTR + F + I + I + R7) according to a Bayesian information criterion (BIC) score of 8,944,815.825 and an Akaike information criterion (AIC) score of 8,943,731.058 to build the phylogenetic tree. The phylogeny of 37 reference strains is shown in Fig. 4 and represented seven *H. pylori* populations: hspEAsia (F16, F30, F32, F57, 51, 52, 83, XZ274, and 35A), hspAmerind (Puno135, Puno120, v225d, Shi112, Cuz20, Shi470, Sat464, Shi417, Shi169), hybrid hspAmerind/hspEAsia (PeCan4), hpAsia2 (SNT49, India7), hpEurope (HPAG1, 26695, B8, Lithuania75, G27, P12, B38, HUP-B14, SJM180, and ELS37), hpAfrica2 (SouthAfrica7), and hpAfrica1 (Gambia94/24, J99, and Pecan18). We assigned strains CHC155 and VN1291 to the hspEAsia population according to clustering with the hspEAsia group strains (Fig. 4).

Virulome and antimicrobial resistance genes of strains CHC155 and VN1291.

1. Virulome

Virulence genes were identified in strains CHC155 and VN1291 by screening the virulence factor database using ABRICATE (Supplementary Table S2). These virulence factors were diverse and classified into eight categories: acid resistance, adherence, endotoxin, molecular mimicry, chemotaxis and motility, proinflammatory effects, toxins, and secretion systems (Supplementary Table S2). Strains CHC155 and VN1291 possessed 19 and 20 kinds of the outer membrane protein genes, *hopA-Z*, among which were *hopH* (*oipA*), *hopS* (*babA*)/*hopT* (*babB*), and *hopU* (*babC*). Interestingly, for some outer membrane proteins, two copies (*hopD, hopH, hopJ, hopZ*) or multiple copies (*hopN, hopO*) were found in one or both genomes (Supplementary Table S3). There was a slight difference in the number of outer membrane proteins (*hopD, hopMN,* and *hopO*) between the two strains. Strain VN1291 carried two genes encoding the HopD protein while strain CHC155 carried only one. Meanwhile, there were three genes encoding HopO proteins in strain VN1291 but only two in strain CHC155.

**Figure 5:**
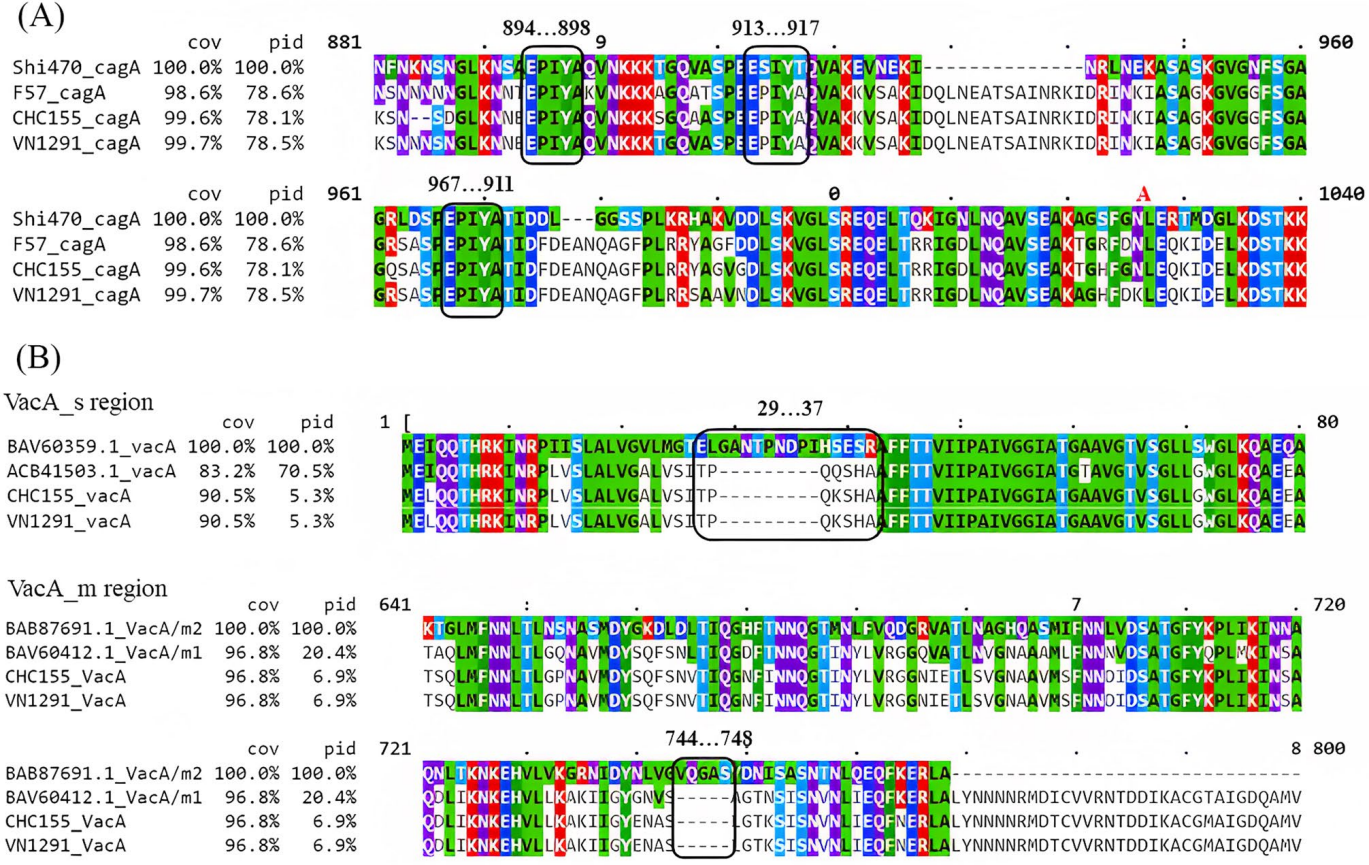
CagA type and VacA type CHC155 and VN1291 strains. (**A**) The EPIYA motifs (three black boxes) and neighboring regions in the C-terminal region of the CagA protein from four strains: Shi470, F57, CHC155, and VN1291. CagA of either strain CHC155 or VN1291 is an ABD type. (**B**) Characterization of VacA in strain CHC155. The black boxes in the figure display the alignment of VacA protein sequences of strain CHC155 and VN1291 compared with the signal region, *s1* (ACB41503.1)*/s2*(BAV60359) (upper box), and intermediate region, *m1*(BAV60412.1)*/m2* (BAB87691.1) (bottom box). The VacA alleles are characterized by deletions in the s- and m-regions corresponding to *s1* and *m1* types.

CagA, an effector protein, and VacA, which encodes a vacuolating and pore-forming protein, were also identified in the two strains. The C-terminus of CagA contained three Glu-Pro-Ile-Tyr-Ala (EPIYA) motifs with neighboring sequences corresponding to ABD-type and East Asian specific CagA-type strains (Fig. 5A), which may undergo tyrosine phosphorylation to hijack internal signaling pathways. In addition, the *vacA* gene motif of CHC155 and VN1291 was assigned to s1m1 (Fig. 5B), which induces a high level of vacuolation and cytotoxicity and is associated with gastric inflammation, peptic ulcer, and gastric cancer^33,34^.

2. Antimicrobial resistance gene profiling

Strain CHC155 was phenotypically resistant to clarithromycin, levofloxacin, and metronidazole but susceptible to amoxicillin and tetracycline (Table 3). Consistently, corresponding resistance mutations in the genes, *gyrA* (HP0701), 23S *rRNA*, and *rdxA* (HP0954) were identified in its genome, whereas no relevant mutations were noted in genes, *pbp1a* (HP0597) or 16S *rRNA* (Table 3). In strain VN1291, we identified resistance mutations in *pbp1A* (HP0597), *rdxA* (HP0954), and 23S *rRNA*. The high sequence coverage of the VN1291 and CHC155 genomes makes variant calling of antimicrobial resistance mutations reliable. To predict antibiotic resistance, we screened the CHC155 and VN1291 genomes using several antimicrobial resistance databases. However, no acquired antibiotic resistance genes were predicted using CARD, Plasmidfinder, ARG-ANNOT, and ResFinder (Supplementary Table S4). However, MEGARes detected a multidrug efflux transporter gene (HP0313) (Supplementary Table S4–6). This gene belongs to the YbfB/YjiJ family of major facilitator superfamily transporters, which is widely distributed in microbial genomes and exhibits a large spectrum of substrate specificities^35^.

**Table 3:** Genetic determinants of antibiotic resistance in *H. pylori* CHC155 and VN1291.

Antibiotic	Minimum inhibitory concentration (mg/L)	Phenotype	Gene	Mutation site
(a) Strain CHC155				
Amoxicillin	0.06	S	*pbp1* (HP0597)	N.D
Clarithromycin	4	R	2 copies of 23S *rRNA*	A2147G
Levofloxacin	8	R	*gyrA* (HP0701)	N87K
Metronidazole	32	R	*rdxA* (HP0954)	T31E, H53R, D59N, Q65^*^
			*frxA*	N.D
Tetracycline	0.12	S	2 copies of 16S *rRNA*	N.D
(b) Strain VN1291				
Amoxicillin	0.5	R	*pbp1* (HP0597)	F366L, S414R, K464insE, D473E
Clarithromycin	4	R	2 copies of 23S *rRNA*	A2147G
Levofloxacin	0.5	S	*gyrA* (HP0701)	N.D
Metronidazole	> 256	R	*rdxA* (HP0954)	Frameshifted, insertion/deletion at approximately position 1,114,371
			*frxA*	N.D
Tetracycline	0.125	S	2 copies of 16S *rRNA*	N.D

*S* Susceptible, *R* resistant. Determined by cut off value according to the European Committee on Antimicrobial Susceptibility Testing (EUCAST) clinical breakpoint (2021-01-01). S is equal to or less than the cut-off value and R is more than the cut-off value. The cut offs (mg/L) were; amoxicillin (0.125), levofloxacin (1), clarithromycin (0.25), tetracycline (1), and metronidazole (8). *N.D.* not detected.

### In vitro assessment of CHC155 and VN1291 virulence

**Figure 6:**
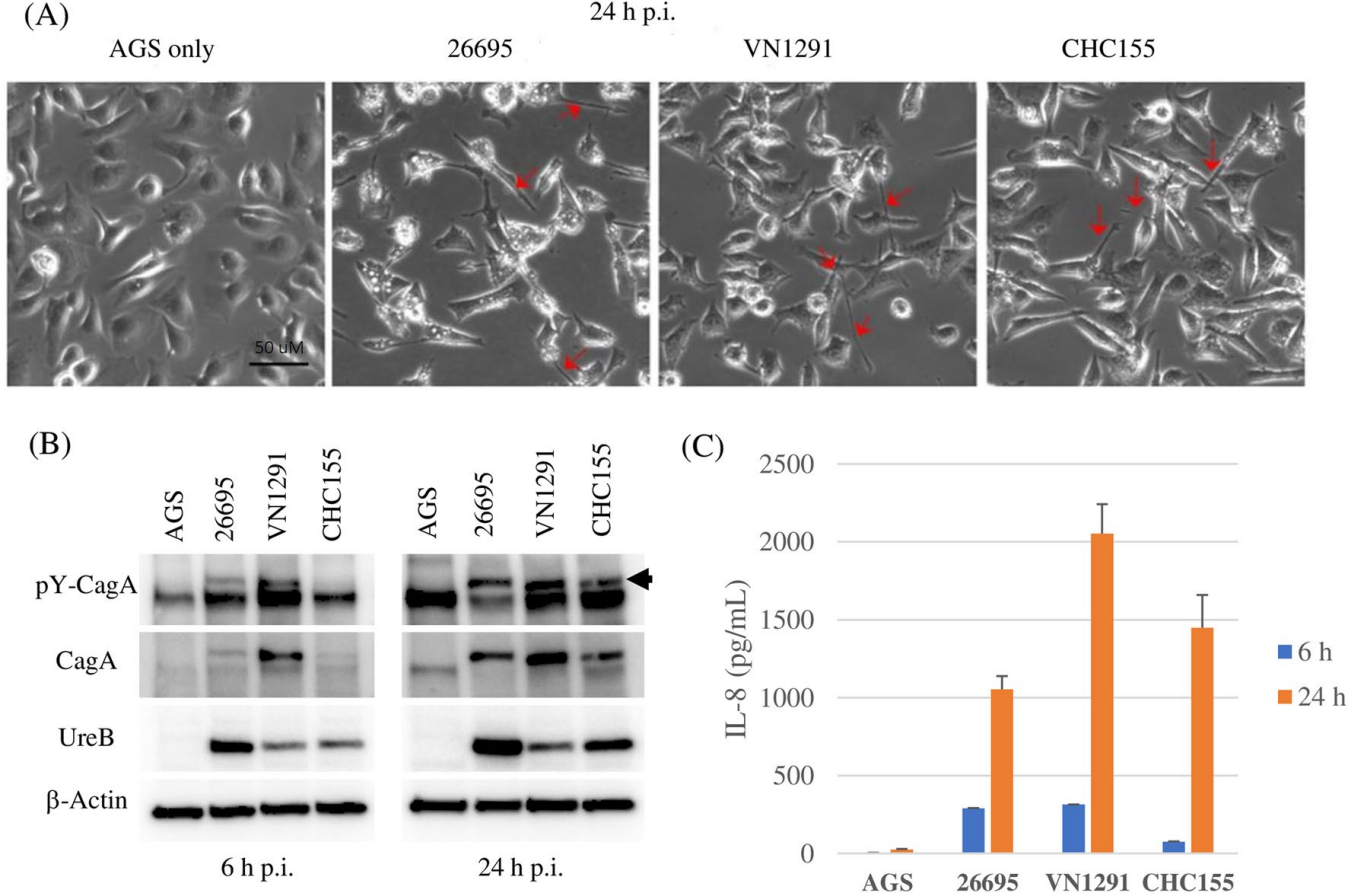
*H. pylori* CHC155 and VN1291 induced the hummingbird phenotype and IL8 secretion in human gastric epithelial AGS cells. AGS cells were incubated with or without *H. pylori* CHC155, VN1291, or 26695 (multiplicity of infection 100) for 6 and 24 h. Bar: 50 μm. (**A**) Cells 24 h after infection. Red arrows indicate cells with the hummingbird phenotype. (**B**) Immunoblot analysis of phosphorylated CagA (pY-CagA), CagA, UreB and β-actin. (**C**) Induction of IL8 during infection (n = 4).

AGS gastric epithelial cells were infected with CHC155 and VN1291 strains to understand their virulence. A cell elongation phenotype, known as the hummingbird phenotype, is observed after CagA injection from *H. pylori* into AGS cells^36–38^. Moreover, induction of IL8 during *H. pylori* infection depends on the presence of *cag*PAI, both in vitro and in vivo^39,40^. To confirm whether such virulent phenotypes occur with these two Vietnamese strains, AGS cells were infected with CHC155 or VN1291, or with 26695 as a control. Twenty-four hours after infection, we observed hummingbird phenotypes in AGS cells (red arrows in Fig. 6A).

After infection of AGS cells with each strain, CagA phosphorylation (Fig. 6B) was assessed in comparison with total CagA by immunoblotting. CagA phosphorylation was induced by strains 26695 and VN1291 at 6 h after infection and the degree of phosphorylation was increased at 24 h. This phenomenon was also observed for strain CHC155, but over a longer time scale; phosphorylation was faint at 6 h, but strong at 24 h. Bacterial counts were monitored using urease subunit UreB, an abundant *H. pylori* protein (Fig. 6B, and Supplementary Fig. S1).

Twenty-four hours after infection, strain CHC155 induced approximately 1.5 times and strain VN1291 approximately 2 times higher levels of IL8 than strain 26695 (Fig. 6C). The level of IL8 secretion indicates the ability to induce inflammatory activity; therefore, strains CHC155 and VN1291 are inflammatory strains.

## Discussion

We report the complete genome of *H. pylori* strains CHC155 and VN1291, which were isolated from Vietnamese patients with non-cardia gastric cancer and duodenal ulcer, respectively. Both strains can induce the hummingbird phenotype, IL8 secretion, and CagA phosphorylation. This indicates that the strains have the potentials to initiate pathogenic changes in the gastric mucosa. Phylogenetic analysis assigned the two strains to the hspEAsia population. In addition, the outer membrane protein compositions of strains CHC155 and VN1291 contain two *hopH(oipA)*, two *hopS(babA)/hopT(babB)*, two *hopJ*, and multiple *hopMN* proteins, which is similar to other *hspEAsia* strains described by Kawai et al*.*^41^. Divergence in the number of outer membrane protein loci between hspEAsia, hspAmerind, hspWAfrica, and hpEurope populations has been shown, and the hpEurope and hspWAfrica populations possess one *hopH* locus and three *babA/babB/babC* loci^41^. It is interesting that we found *hopU (babC)* from both of Vietnamese strains in low coverage to it from strain 26695. The differences in the number of outer membrane proteins and in genetic variation between two strains of the same population but isolated from two distinct diseases, cancer and duodenal ulcer, and between *H. pylori* populations, may reflect a flexibility and adaptation capability of the *H. pylori* genome in host interaction.

Some *H. pylori* virulence factors are crucial for prolonging infection in the gastric mucosa via molecular mimicry. Strain CHC155 harbors *futB* and *futC*, which encode Lewis antigens expressed in the gastric mucosa (Supplementary Table S2). This antigenic mimicry suppresses an immune response against the bacteria and allows it to adhere to the gastric mucosa^42^. NapA promotes adhesion of human neutrophils to endothelial cells and the production of reactive oxygen radicals. In addition, the outer membrane family proteins, *babA/babB* and *hopQ* are involved in *H. pylori* adhesion^43^. The inflammatory effect of *H. pylori* is associated with the effect of *oipA* protein (*hopH*) on IL8 production^44^.

Our interest focused on important known virulence factors of *H. pylori* that are associated with gastroduodenal diseases. Strains CHC155 and VN1291 harbored the EastAsian-type *cagA* (ABD motif) and *vacA* s1m1. Both strains also possessed *cag*PAI, a genomic island that encodes the T4SS machinery for translocating CagA into host cells. In addition, strain CHC155 possessed two *tfs3* ICEs that contain a complete T4SS cluster. The genetics of *tfs3/4* ICE varies among *H. pylori* strains; they can harbor a complete or partial *tfs* fragment, or no *tfs*^22,23^. Moreover, *tfs* ICE is frequently exchanged and integrated into the genome in a hybrid *tfs3-4* ICE through conjugation^22,23^. Compared with previous genome studies of *H. pylori*^22,23,45,46^, we found that strain VN1291 possessed one *tfs3* and a hybrid *tfs3-4*. Furthermore, we identified a KHP30-like prophage in the genome of strain CHC155 and a KHP40-like prophage in strain VN1291. The prophage was integrated between *comGF* at the 5′ end and a putative outer membrane protein at the 3′ end, similar to the other *hspEAsia* strains^47^. *comGF* plays a role in transformation and DNA binding, which contributes to the genetic variability of *H. pylori*^48^, while outer membrane proteins mediate adherence to the gastric epithelium and are associated with the clinical outcome of the infection^49^. These findings indicate that the prophage genetic element is adaptable in the *H. pylori* population.*cagA* gene*-*positive strains affect the severity of gastroduodenal disease. The phosphorylated or non-phosphorylated form of CagA activates downstream host cell-signaling pathways by binding to adaptor proteins, such as Crk, Grab2, HSP-2, PAR1, and c-Met^50,51^. Crk-CagA interaction induces cell–cell dissociation and development of the hummingbird phenotype^50,51^. Therefore, our in vitro results of CagA phosphorylation and the hummingbird phenotype in infected AGS cells by strain CHC155 or VN1291 indicate the potential virulence of both of strains. Interestingly, we observed higher levels of CagA and phosphorylated CagA in strain VN1291 at 6 h compared with levels in other strains, and increased levels of CagA at 24 h post infection in strain CHC155 compared with that in strain 26695, even if we take into account the strain specificity of the commercial CagA antibody used in our immunoblot analysis, which was originally generated using Western-CagA epitopes of a Western *H. pylori* strain. Regulation of *cagA* transcription by NaCl, was found in strain 26695 and Colombian clinical isolates, which is mediated via two copies of a TAATGA motif in the CagA promoter region^52^. Moreover, a + 59 motif in the *cagA*-5′-untranslated region influences the levels of CagA^53^. Further investigations are necessary to understand this mechanism of CagA regulation in Vietnamese strains.

The observation of high IL8 levels following infection with strains CHC155 and VN1291 compared with strain 26695 was particularly interesting, although a difference in IL8 levels between *cag*PAI-positive strains was previously observed^54^. Two main hypotheses can account for this difference. First, IL8 secretion may be induced when *tfs3* ICE is present, as is the case for *cag*PAI^55^. The genome analysis showed that strains CHC155 and VN1291 possessed a complete T4SS cluster (11 T4SS core genes) in the *tfs3* ICE, whereas *virB8*, *virB9*, *virB10*, *virB11,* and *virD4* genes were absent in strain 26695. Second, the effector protein *ctkA* in *tfs3* ICE might promote proinflammatory activity. Strains CHC155 and VN1291 both possess *ctkA* but strain 26695 does not. Recent evidence indicates that T4SS genes of *tfs3* support the injection of CtkA into host cells and the induction of high levels of IL8 secretion^56^. Our virulence profiling showed that strain VN1291 is very similar to strain CHC155. This may be because both strains belong to the hspEAsia population, although they were isolated from two patients with different diseases, duodenal ulcer (DU: VN1291) and gastric cancer (GC: CHC155). Our previous phylogenetic analysis at the whole genome scale showed that the indicated DU and GC strains were distributed together^24,57^. In addition, our previous genome wide association study on hspEAsia strains indicated that single nucleotide polymorphisms between strains causing different diseases could be discovered and underlying mechanisms suggested, such as electric charge alteration at the ligand-binding pocket, change in subunit interaction, and mode-switching DNA methylation. The virulent gene components of DU and GC strains were similar and single nucleotide polymorphisms may affect host–pathogen interaction and are novel candidates for disease discrimination^57^.

## Conclusions

Here, we report the complete genomes of strains CHC155 and VN1291, which were isolated from patients with non-cardia gastric cancer and duodenal ulcer, respectively, as two representative virulent strains from Vietnam. Both strains carry East Asian-type *cagA* and *vacA s1m1*. Furthermore, each strain possesses *cag*PAI, two *tfs3/tfs4* ICEs, and a prophage. Strains CHC155 and VN1291 can induce proinflammatory responses and morphological changes in gastric epithelial cells, indicating their potential virulence.

## Materials and methods

### *H. pylori* and genome sequencing

*Helicobacter pylori* CHC155 was isolated from a 61 year-old male patient with non-cardia gastric cancer at Cho Ray Hospital, Ho Chi Minh. *H. pylori* VN1291 was isolated from a 43-year-old female patient with duodenal ulcer at Cho Ray Hospital, Ho Chi Minh. After the international transfer of gastric antral biopsy samples to Oita University, both strains were isolated using standard culture methods as previously described^9^. DNA was extracted using a DNeasy Blood & Tissue kit (Qiagen Inc., Valencia, CA, USA). DNA concentration was measured using a Quantus Fluorometer (Promega, Madison, Wiscosin, USA). The extracted genomic DNA was sheared for library construction using a Covaris g-TUBE device according to the manufacturer’s instructions. High-throughput genome sequencing was performed on a HiSeq 2500 (2 × 150 paired-end reads) for strain CHC155, and MiSeq (2 × 300 paired-end reads) system for strain VN1291, following each of the manufacturer’s instructions (Illumina, San Diego, CA, USA). Trimmomatic v. 0.35 was used to remove adapter sequences and low-quality bases from raw short-read data^58^.

A SMRTbell library was prepared using a SMRTbell template Prep Kit 1.0 (Pacific Bioscience, CA, USA). DNA fragments larger than 17 kb, were selected using the BluePippin system (Saga Science, MA, USA). For each *H. pylori* strain, one SMRT cell was run on the PacBio RS II System with P6/C4 or P6/C4v2 chemistry and 360-min movies (Pacific Biosciences, Menlo Park, CA, USA). SMRT sequencing data were analyzed using SMRT Analysis version 2.3.0 via the SMRT Portal.

### De novo assembly and genome annotation

To generate a complete genome assembly, de novo assembly was performed with the hybrid-assembly method using Unicycler v.0.4.8, which combined both long and short reads with default parameters; command line: unicycler -1 SHORT1 -2 SHORT2 -s UNPAIRED -l LONG –min_fasta_length 200^59^. Briefly, short reads (HiSeq/MiSeq), were assembled into several contigs, and gaps between contigs were connected by long-reads (PacBio in strain CHC155 and Oxford Nanopore in strain VN1291) to generate complete circular contigs, which have one link connecting the end to the start. The assembly was subsequently polished for a maximum of 10 rounds using Pilon^60^ and then Unicycler software was applied. Finally, genome features were annotated using PROKKA v1.14.6^61^, a rapid bacterial genome annotation pipeline, using default parameters.

### Bioinformatics software

The core genome alignment of strains CHC155 and VN1291 and the reference strains was obtained using Roary v3.13.0^62^ with the following parameters: minimum percentage identity of 80% in blastp and core genes in 99% of isolates; command-line: roary –i 80 –e –mafft *.gff. The *H. pylori* reference strains represented in seven populations (hpAfrica2, hpAfrica1, hpEurope, hpAsia2, hybrid hspAmerind/hspEAsia, hspAmerind, and hspEAsia) were assigned in previous studies^63–65^. The high-resolution phylogenetic tree was constructed via a generalized time-reversible (GTR + F + I + I + R7) model to estimate the pairwise distance of 1,036 core genome alignments from the Roary output. These core genome alignments were used to construct phylogenetic trees using the maximum-likelihood method, the GTR model, and IQ-TREE v.1.6.12^66^; command-line: iqtree -T AUTO -m MFP –s core_gene_alignment.fasta. Prophage was predicted using the PHASTER server^67^ and genomic islands were predicted using the IslandViewer4 server with the GenBank files from PROKKA v1.14.6 and strain F57 serving as a reference for genome comparison^68^. The circular genome was visualized using Proksee (https://proksee.ca)^30^ and SnapGene v.6.2.2, and the genetic scheme of genomic islands were plotted using the genoplotR v.0.8.11 package (RStudio, v. 1.3.9, PBC). The virulence and antimicrobial resistance genes of strain CHC155 and VN1291 were retrieved from public databases (VFDB, ResFinder, MEGARes, ARG-ANNOT, CARD, and PlasmidFinder) using ABRICATE v1.0.0^16^.

### Determination of antibiotic resistance phenotypes and genotypes

Antimicrobial susceptibility was assessed using Etest^®^ (bioMerieux) for five antibiotics (amoxicillin, clarithromycin, levofloxacin, tetracycline, and metronidazole) following European Committee on Antimicrobial Susceptibility Testing (EUCAST) v.21.01.01 protocols on Müller–Hinton agar plates supplemented with 5% horse blood. The minimum inhibitory concentrations of the antibiotics were checked every day after incubation for 3–6 days and determined. *H. pylori* strain 26695 was used as a control strain. Clinical breakpoints between resistant and susceptible strains were determined following the EUCAST guidelines available at http://www.eucast.org/.

The genetic determinants of antibiotic resistance in strain 26695, *gyrA* (HP0701), 23S *rRNA*, *rdxA* (HP0954), *pbp1* (HP0597), and *16S rRNA*, were used to retrieve those in strains CHC155 and VN1291 using the blastn algorithm with a minimum coverage of 80% and a minimum identity of 90%. Nonsynonymous mutations in either Vietnamese strain versus 26695, were compared with resistance mutations described in *H. pylori* strains^69^.

### Evaluation of *H. pylori* virulence to and IL8 secretion from AGS cells

The virulence of *H. pylori* strains CHC155, VN1291, and reference strain 26695, was assessed experimentally by infecting human gastric epithelial AGS cells as described previously^70^. AGS cells were originally isolated in 1979 from the stomach tissue of a 54-year-old, white, female patient with gastric adenocarcinoma. These cells exhibit epithelial morphology^71^. The experiments were performed independently twice. Briefly, AGS cells were seeded onto six-well plates, grown overnight in RPMI 1640 medium supplemented with 10% FBS, and incubated at 37 °C in 5% CO_2_. *H. pylori* strains were suspended in RPMI 1640/10% FBS from a 2-day *Brucella* agar plate culture supplemented with 7% horse blood and added to the 70–80% confluent AGS culture at a multiplicity of infection of 100. After co-culture for 6 and 24 h, cells were fixed with 10% paraformaldehyde for 15 min, washed with PBS, and formation of the hummingbird phenotype examined under a phase-contrast microscope in randomly chosen fields. IL8 in the supernatant of infected AGS cells in 12-well plates (n = 4) was measured using a Human IL8 Uncoated ELISA Kit (Invitrogen, USA). Western blot analysis was performed using infected cell lysates from a 12-well plate culture. The antibodies against the following were used: p-Tyr (PY-99, Santa Cruz Biotechnology, Dallas, TX, USA), CagA (Austral Biologicals, San Rarnon, CA, USA), urease B (Institute of Immunology, Tokyo, Japan), and β-actin (Sigma-Aldrich, St. Louis, MO, USA).

### Ethics approval and consent to participate

The study was conducted according to the guidelines of the Declaration of Helsinki and approved by the Ethics Committee of Oita University. Informed consent was obtained from all subjects involved in the study.

### Consent for publication

Written informed consent was obtained from the patients to publish their data in this paper.

## Supplementary Information

Supplementary Tables.

Supplementary Figures.

## Data Availability

The whole genome sequence data of strains CHC155 and VN1921 have been deposited at DDBJ/ENA/GeneBank. The accessions numbers are AP026446 (strain CHC155), AP026444 (strain VN1291), and AP026445 (plasmid pVN1291 in strain VN1291).
