# Supplementary material for: Comparative genomics of two Vietnamese Helicobacter pylori strains, CHC155 from a non-cardia gastric cancer patient and VN1291 from a duodenal ulcer patient
Source: Sci Rep. 2023 May 31;13:8869. doi: 10.1038/s41598-023-35527-4 (PMC10232435; doi:10.1038/s41598-023-35527-4)
Supplement: Supplementary file 2 — Supplementary Figures. [file 41598_2023_35527_MOESM2_ESM.docx]

**Comparative genomics of two Vietnamese *Helicobacter pylori* strains, CHC155 from a non-cardia gastric cancer patient and VN1291 from duodenal ulcer patient**

Bui Hoang Phuc^1,2^, Vo Phuoc Tuan^3^, Tran Thanh Binh^3^, Pham Huu Tung^3^, Tran Dinh Tri^3^, Ho Dang Quy Dung^3^, Ngo Phuong Minh Thuan^3^, Kartika Afrida Fauzia^1^, Evariste Tsinbangu Kabamba^1,4^, Ricky Indra Alfaray^1^, Batsaikhan Saruuljavkhlan^1^, Takashi Matsumoto^1^, Junko Akada^1^, Yoshio Yamaoka^1,5^*

**Supplementary Information 2**

**Figure S1**. Quantification of immunoblotting analysis of CagA and phosphorylated CagA (pY-CagA), per UreB (control for bacterial amount) showing in **Figure 6 B**.

**Original western blots and the membrane used in Figure 6B**

1. **(B)**

**Figure S1**. Quantification of immunoblotting analysis of CagA and phosphorylated CagA (pY-CagA), per UreB (control for bacterial amount) showing in **Figure 6 B**. Quantification was done using Image Lab Software 6.1 (Bio-Rad).

**Original western blots and the membrane used in Figure 6B**

**Anti-phospho-tyrosine pY99 (to show pY-CagA)**

**
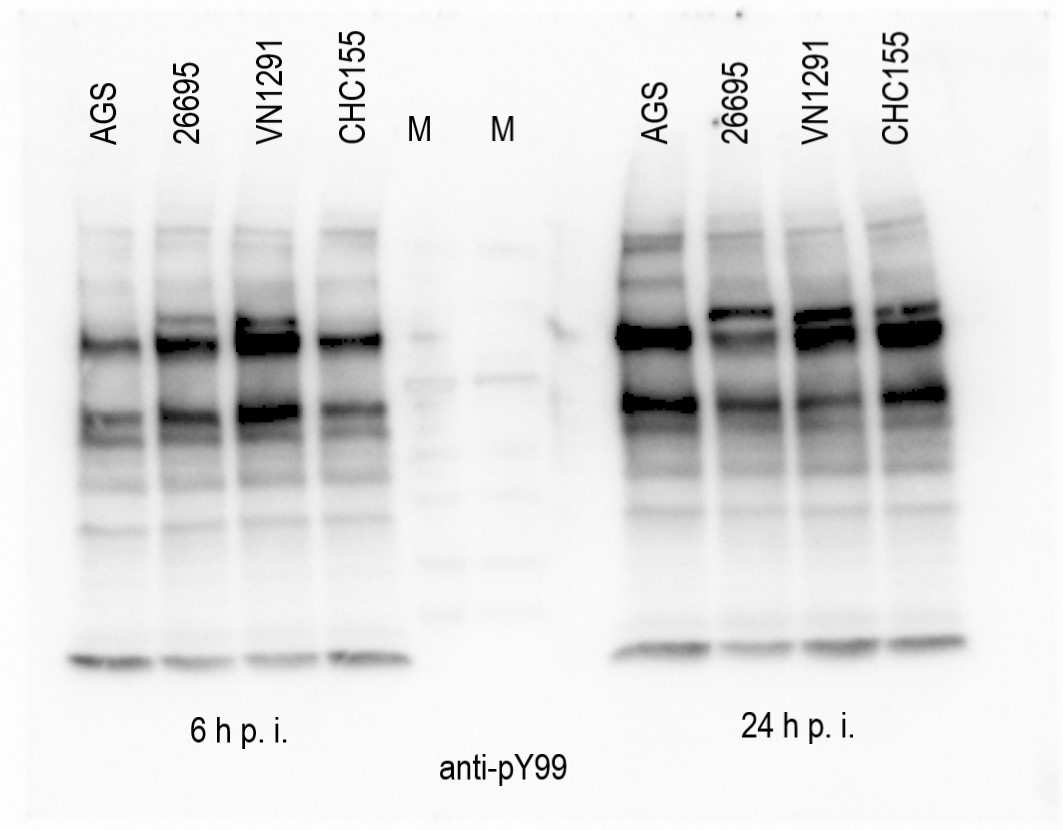
**

**Anti-CagA**


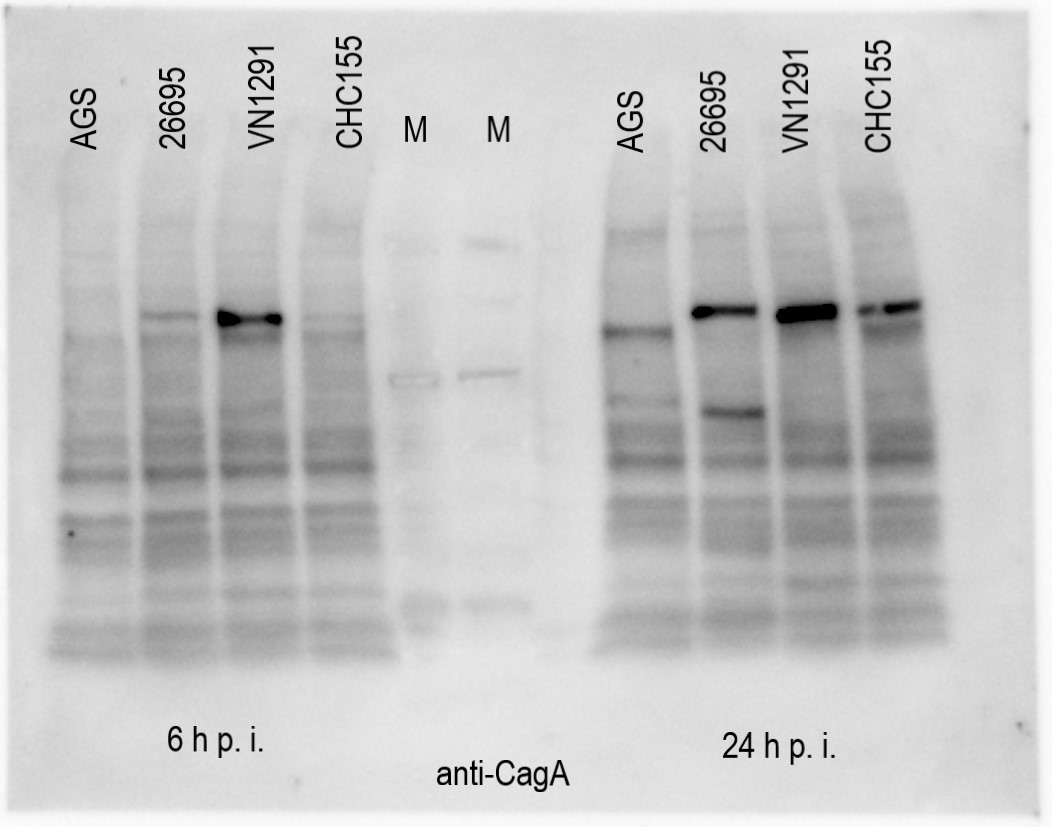


**Anti-β-Actin**


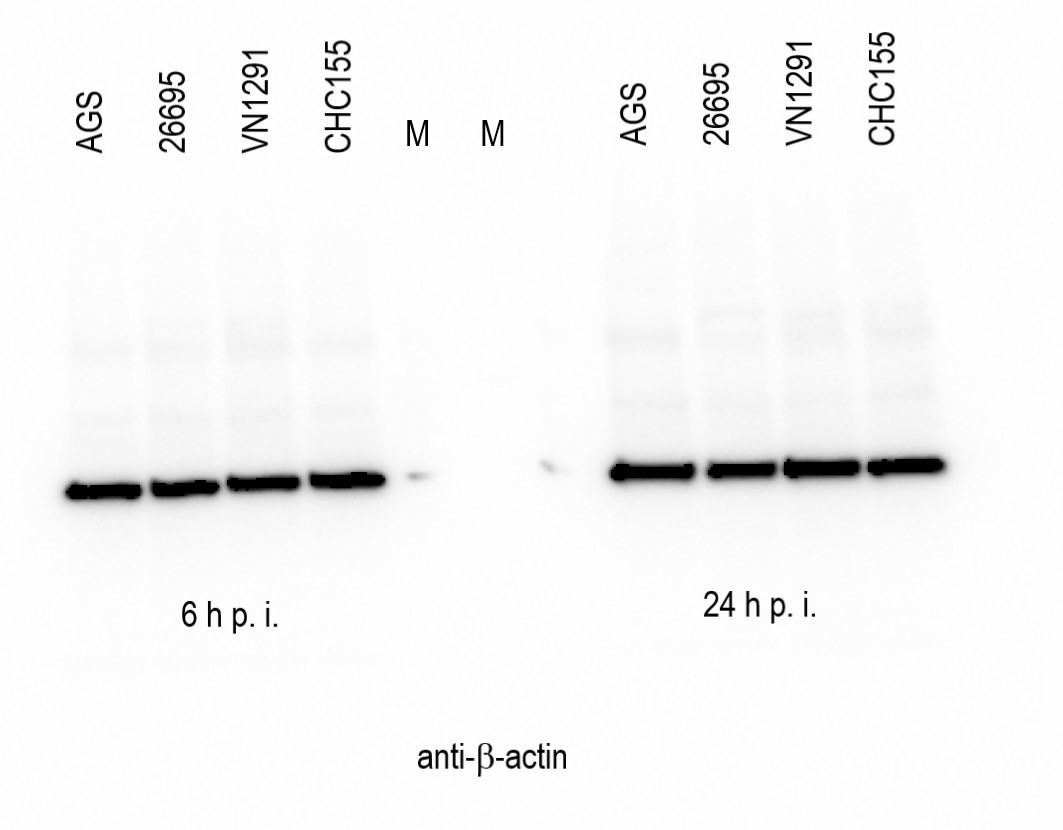


**Anti-UreB**

**
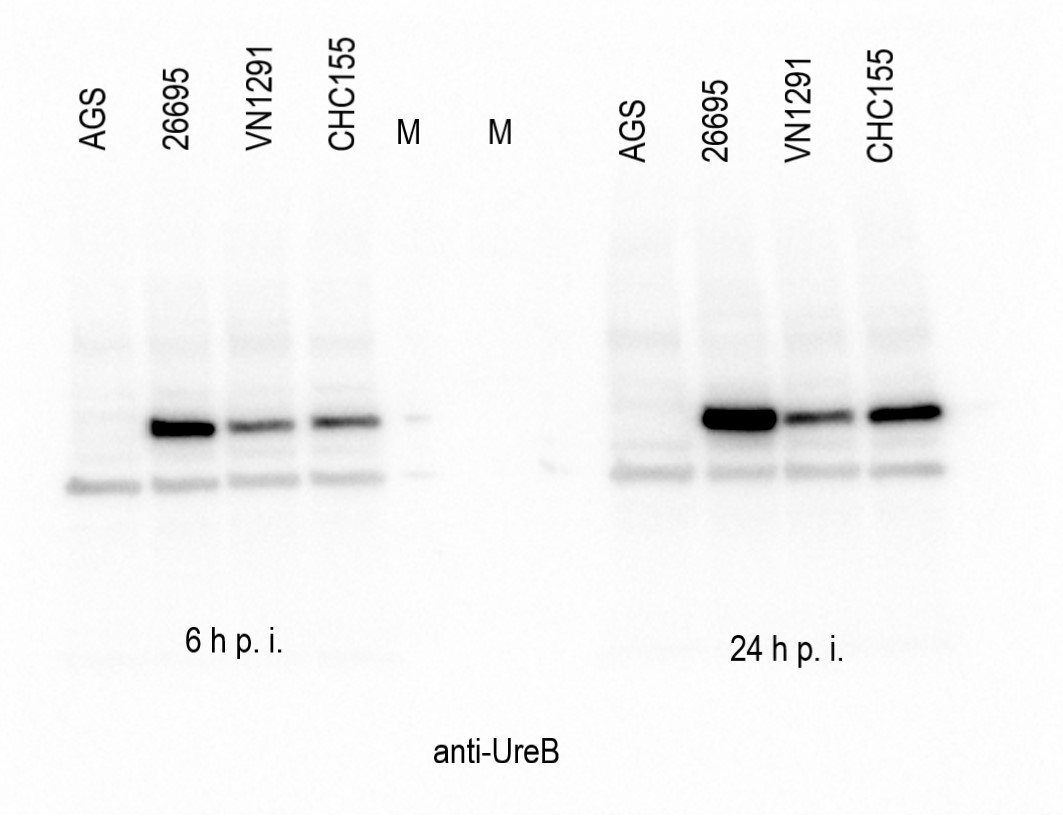
**

**Protein blotted PVDF membrane used for Western blotting analysis**


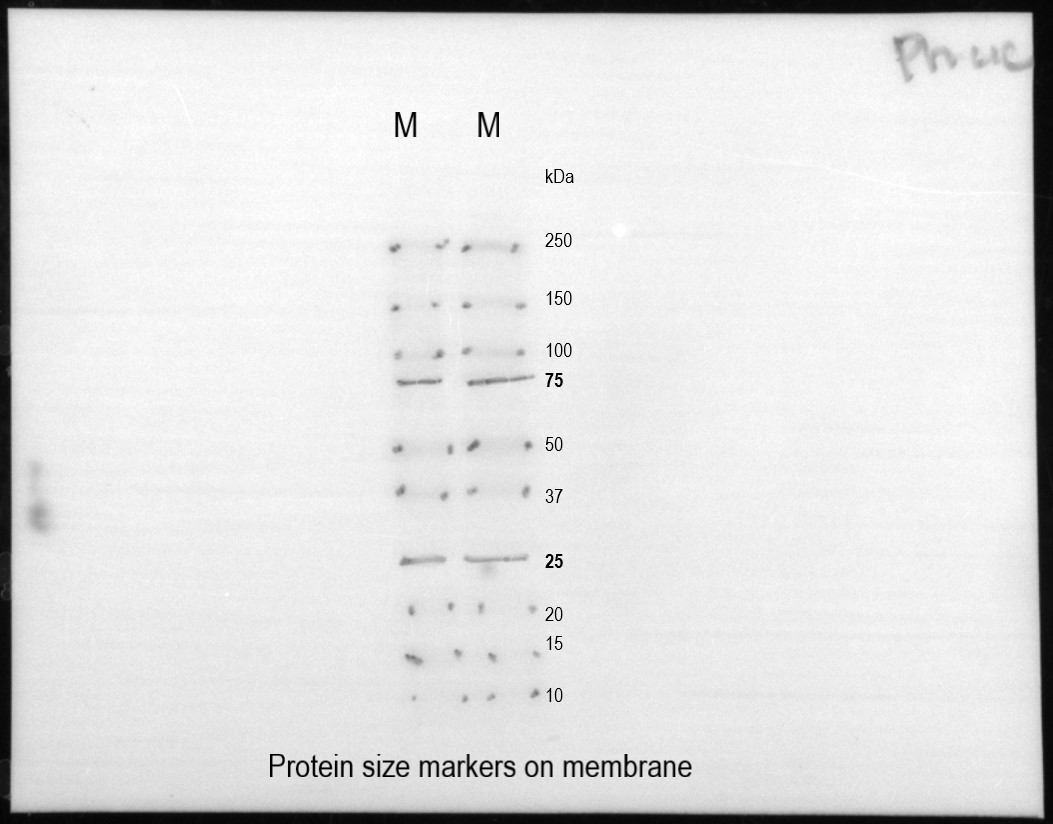
Size of markers (Central two lanes) were showing as 10 marker bands drown as dots and lines by pencil on membrane just after blotting of the SDS-PAGE. All Western blotting image detection were done using this membrane in order of presented.
